# Implications and Economic Impact of Applying International Guidelines and Recommendations to the Management of High-Risk Group of Type 2 Diabetes Mellitus Patients in India

**DOI:** 10.7759/cureus.22141

**Published:** 2022-02-11

**Authors:** Zeenat Fatima, Shubham Atal, Rajnish Joshi, Balakrishnan Sadasivam

**Affiliations:** 1 Medical Affairs, Novo Nordisk, Bhopal, IND; 2 Pharmacology, All India Institute of Medical Sciences, Bhopal, IND; 3 Internal Medicine, All India Institute of Medical Sciences, Bhopal, IND

**Keywords:** treatment guidelines, cost effectiveness, cardiovascular outcomes, sglt-2 inhibitors, t2dm

## Abstract

Objectives

Sodium-glucose cotransporter-2 (SGLT-2) inhibitors and dipeptidyl peptidase IV (DPP-IV) inhibitors are recommended as preferred add-on oral antidiabetic drugs (OADs) after metformin among type 2 diabetes mellitus (T2DM) patients with atherosclerotic cardiovascular disease (ASCVD), heart failure (HF), and chronic kidney disease (CKD). They are generally many folds costlier than other OADs. This is a simulatory analysis to assess the incremental cost escalation and risk reduction with their hypothetical substitution/addition in prescriptions of high-risk patients.

Methods

A simple simulation of cost-effectiveness analysis was performed using prescriptions of T2DM patients with established cardiovascular (CV) or renal disease or high-risk factors. SGLT-2 and DPP-IV inhibitors with proven benefits/safety were substituted or added in place of other OADs. Increments in treatment costs were calculated, and the anticipated decrease in hazards was extrapolated from cardiovascular outcome trials (CVOTs) and real-world studies. The incremental cost-effectiveness ratios (ICERs) were calculated.

Results

Prescriptions of 351 patients with a mean age of 58.04 ± 8.67 years were analyzed. The median annual acquisition cost of drug therapy for diabetes per patient was found to be Indian national rupee (INR) 8,964.4 for the original prescriptions when calculated using median retail prices of drugs prescribed for diabetes. Upon substituting one of the SGLT-2 inhibitors for the other OADs in the regimen, the cost increased to INR 12,265 (increase by 36.8%) for dapagliflozin, and INR 26,718 and INR 29,419 (increase by ~200%), respectively, for canagliflozin and empagliflozin. Upon calculating the ICERs, additional cost to prevent one all-cause death with dapagliflozin substitution is INR 660,020-25,384,369; INR 2,223,326 with empagliflozin substitution and INR 8,069,818 with canagliflozin substitution. The ICER for prevention of hospitalization with HF with dapagliflozin substitution is INR 1,320,040-1,435,543; INR 4,010,706 with empagliflozin and INR 5,548,000 with canagliflozin. To prevent a three-point major adverse cardiac event (3P-MACE), INR 2,062,562 would be needed with dapagliflozin substitution, and INR 3,146,861 and INR 3,859,478 with empagliflozin and canagliflozin, respectively. Incremental costs for various outcomes were higher with the addition of SGLT-2 inhibitors and significantly more if substitution with sitagliptin/linagliptin was also done. The numbers needed to treat were calculated too and ranged from 35 to 1,831 for various outcomes and drugs.

Conclusion

While the recommendations for use of SGLT-2 and DPP-IV inhibitors are adequately backed by evidence from CVOTs and real-world data, the incremental costs per event reduction are quite high for most outcomes in the Indian context. Dapagliflozin, being available as cheaper generic versions, appears to be most effective for most outcomes. Interpretations are subjective in terms of value assigned for preventing a major event.

## Introduction

Various international and regional guidelines like the American Diabetes Association (ADA) Standards of Medical Care in Diabetes (USA), European Association for the Study of Diabetes (EASD)/European Society of Cardiology (ESC), American Association of Clinical Endocrinologists and American College of Endocrinology (AACE/ACE), National Institute for Health and Care Excellence (NICE) (UK), Indian Council of Medical Research (ICMR) (India) have been recommending the use of sodium-glucose cotransporter-2 (SGLT-2) inhibitors as the preferred add-on oral therapy, alongside injectable glucagon-like peptide 1 receptor agonists (GLP-1 RAs), after metformin among type 2 diabetes mellitus (T2DM) patients with atherosclerotic cardiovascular disease (ASCVD), heart failure (HF), and/or chronic kidney disease (CKD), mainly due to their proven mortality and cardiovascular/renal outcome benefits as well as safety shown in cardiovascular outcome trials (CVOTs). Similarly, dipeptidyl peptidase IV (DPP-IV) inhibitors are also recommended as one of the alternative preferred add-on drugs to minimize chances of hypoglycemia and avoid weight gain wherever needed, along with proven cardiovascular (CV) safety [1-5].

In fact, the latest guidelines from the ADA (2022) have now changed the recommendation to state that GLP-1 RAs and SGLT-2 inhibitors, with or without metformin, “are appropriate initial therapy for individuals with T2DM with or at high risk for atherosclerotic cardiovascular disease, heart failure, and/or chronic kidney disease” [1]. The guidelines maintain that a patient-centered approach should guide the choice of pharmacologic agents. They explicitly mention that drugs with proven CV benefits should be used in patients with specific indicators of high ASCVD risk - age ≥ 55 years with more than 50% stenosis of coronary, carotid, or lower extremity stenosis or left ventricular hypertrophy (LVH) [1]. There are no such explicit recommendations in these guidelines for T2DM individuals without established ASCVD and having multiple recognized risk factors, such as hypertension, dyslipidemia, obesity, smoking, and family history of diabetes, that are known to substantially increase the risk of CV complications and worsen outcomes, but SGLT-2 and DPP-IV inhibitors are listed as the preferred oral antidiabetic drugs (OADs) as an add-on to metformin generally wherever feasible.

In 2019, an Asian perspective and expert recommendation [6] was published, which reviewed and consolidated the evidence for SGLT-2 inhibitors from CVOTs, large-scale real-world evidence (RWE) studies, as well as clinical trials in Asian populations, and recommended that SGLT-2 inhibitors should be considered early in the treatment of T2DM patients with multiple risk factors, as the drugs have multifactorial effects on blood pressure, weight, insulin sensitivity, and albuminuria. These benefits, along with control of other risk factors, are known to confer protection from the development of CVD.

These classes of drugs are relatively expensive in India, particularly the SGLT-2 inhibitors, as most of them are under patent protection. As compared to drugs like sulfonylureas (SUs) and pioglitazone, daily therapy with any SGLT-2 inhibitor or DPP-IV inhibitor with proven CV/renal benefit or safety is many folds costlier. T2DM prescriptions in India are written with particular emphasis on cost considerations leading to the relatively less frequent prescription of DPP-IV inhibitors or even lesser of SGLT-2 inhibitors, as shown in multiple prescription studies [7-9]. Other OADs also have issues related to weight and hypoglycemia, with no evidence of beneficial CV or renal effects of these drugs.

The prevalence of cardiovascular disease (CVD) including HF, myocardial infarction (MI), and stroke is quite high (21-32%) globally, and in Asia and India, among individuals with T2DM, making prevention of CV complications a very important objective in the management of diabetes [10]. However, the cost of diabetes treatment is already very high and concerns have been raised regarding the financial burden it puts especially on low-income Indian families who may have to spend as much as 25% of their income on diabetic care [11]. This needs to be weighed against the additional costs that are associated with managing CV and renal complications, which creates a situation of a dilemma for prescribers as well as patients. So, this study has been conducted as a basic pharmacoeconomic simulatory analysis to assess the cost escalation and risk reduction, with additional safety, which would be expected if SGLT-2 and DPP-IV inhibitors were hypothetically substituted as add-on drugs in prescriptions of T2DM patients with ASCVD, HF, and/or CKD or those who are clinically judged to be at high risk for the same due to presence of multiple risk factors.

## Materials and methods

This is a partial pharmacoeconomic evaluation - a cost-effectiveness analysis with a simple and practical simulation using primary and secondary data. From the research database of diabetes prescription records available with us for T2DM patients coming to the diabetes specialty clinic at our tertiary care center in the last one year, we identified the prescriptions of high-risk T2DM individuals, those with a history of coronary artery disease (CAD), HF, nephropathy, and/or stroke, and all prescriptions in which multiple medications like antihypertensives, statins, and antiplatelets were given.

After identifying the patients in these disease/risk categories, we added or substituted SGLT-2 inhibitors, with or without an additional substitution of DPP-IV inhibitor in place of other OADs in the original prescription, if drugs with proven benefits/safety from these classes were not already prescribed. For addition/substitution, we used drugs with proven benefits or established CV/renal safety, which are generally prescribed otherwise at our center (empagliflozin, dapagliflozin, canagliflozin, linagliptin, and sitagliptin) at maximal doses. Metformin and insulins were not substituted wherever prescribed. Essentially, the substitutions involved replacing SUs, thiazolidinediones (TZDs), and DPP-IV inhibitors like teneligliptin and vildagliptin with empagliflozin/dapagliflozin/canagliflozin or sitagliptin/linagliptin wherever applicable, to compare the resulting costs with SGLT-2 inhibitor addition too.

The escalation in costs of drug treatment (additional drug costs) was calculated, and the anticipated gain in outcomes was analyzed by simulating data from available CVOTs and one additional large RWE study. The incremental cost-effectiveness ratio (ICER) was calculated as follows:

ICER = Average additional cost with SGLT-2 inhibitors substitution or addition (± DPP-IV inhibitor)/Difference in hazards rates.

For cost escalation calculation, the average retail prices of the drugs were calculated from a recognized commercial drug directory, i.e., Current Index of Medical Sciences (CIMS) India, September-December 2021. The costs were expressed as median (and interquartile range) due to high variations in the drug prices. The median annual cost (per patient) was calculated. The data on anticipated benefit were extrapolated from published trials and studies in terms of reduction in CV events (expressed as per 1,000 patient-years), as measured over different follow-up periods from the studies. The extrapolation is justified on the basis of representation of the Asian population in the CVOTs and RWE, and consistency of results when tested for heterogeneity by race.

## Results

Patient population and antidiabetic drug regimens

A total of 351 prescriptions written for patients with T2DM with established CVD/renal disease/HF (21.4%) or with multiple risk factors were analyzed. Table 1 shows the demographic and disease characteristics of the patient population studied. Mean age was 58.04 ± 8.67 years, with a predominance of males (54.4%), mean duration of diagnosed diabetes of 8.53 ± 3.83 years, and a mean BMI of 26.56 ± 3.38. More than 20% of individuals had a duration of disease of more than 10 years. Hypertension (71.8%) was the most common comorbidity, followed by dyslipidemia (38.17%) and hypothyroidism (8.5%). Nephropathy (9.7%) was the most common complication, followed by CAD (8.3%) and neuropathy (5.4%) (Table 1).

**Table 1:** Demographic and disease characteristics.

Characteristics (N = 351)	Frequency/proportion (%) or mean ± SD
Age (years)	
Overall	58.04 ± 8.67
Males	58.35 ± 8.55
Females	57.71 ± 8.82
Gender	
Males	191 (54.4%)
Females	160 (45.6%)
Duration of diagnosed diabetes (years)	
Overall	8.53 ± 3.8
Males	8.59 ± 3.7
Females	8.46 ± 3.99
BMI (in kg/m2)	26.56 ± 3.38
Males	27.26 ± 3.29
Females	26.38 ± 3.58
Complications	
Nephropathy	34 (9.69%)
Coronary artery disease	29 (8.26%)
Neuropathy	19 (5.41%)
Heart failure	12 (3.42%)
Comorbidities	
Hypertension	252 (71.8%)
Dyslipidemia	134 (38.17%)
Hypothyroidism	30 (8.5%)
History	
History of smoking	44 (12.54%)
Family history of diabetes	180 (51.28%)

A total of 986 drugs were prescribed for diabetes including insulin with a mean of 2.8 ± 1.14 antidiabetic drugs prescribed per prescription (n = 351). Table 2 shows the pattern of regimens and drugs that the patients were prescribed. Most patients were on dual (30.76%) and triple (28.77%) drug regimens. After metformin, DPP-IV inhibitors like teneligliptin and vildagliptin were most commonly prescribed (21.1% prescriptions) while SGLT-2 inhibitors like dapagliflozin and empagliflozin were prescribed in only 4.27% (n = 15) of prescriptions. Insulin preparations were prescribed in 18% of prescriptions. Based on currently prescribed regimens, it was found that 12.25% (n = 43) of prescriptions did not require any addition/substitution (Table 2).

**Table 2:** Pattern of drugs and regimens prescribed. DPP-IV = dipeptidyl peptidase IV; SGLT-2 = sodium-glucose cotransporter-2.

Drug regimens/drugs prescribed	Frequency (%)
Regimens	
Single drug (1)	44 (12.5)
Dual drug (2)	109 (30.8)
Triple drug (3)	101 (28.8)
Quadruple drug (4)	75 (21.4)
≥5	23 (6.5)
Drugs	
Insulin	63 (17.9)
Metformin	310 (88.3)
DPP-IV inhibitors	184 (52.4)
Teneligliptin	94 (26.8)
Vildagliptin	60 (17.1)
Sitagliptin	27 (7.7)
Linagliptin	3 (0.09)
SGLT-2 inhibitors	15 (4.3)
Dapagliflozin	10 (2.8)
Empagliflozin	5 (1.4)

Costs of drug acquisition per prescription

Table 3 shows the median anti-diabetic drug acquisition costs for the prescriptions written - original cost as well as with the hypothetical substitution/addition of drugs (Table 3).

**Table 3:** Direct (drug acquisition) costs of prescriptions for diabetes. * INR = Indian national rupee (1 USD = ~INR 75); IQR = interquartile range.

S. No	Prescription type (N = 351)	Median annual cost (IQR) in INR*
1.	Original as prescribed	8,964.4 (17,886.8)
2.	Dapagliflozin substitution	12,264.5 (16,167.31)
3.	Empagliflozin substitution	29,419 (16,167.31)
4.	Canagliflozin substitution	26,718 (16,167.31)
5.	Dapagliflozin and sitagliptin/linagliptin substitution	24,102.4 (19,493.2)
6.	Empagliflozin and sitagliptin/linagliptin substitution	41,257.4 (19,493.2)
7.	Canagliflozin and sitagliptin/linagliptin substitution	38,556.4 (19,493.2)
8.	Dapagliflozin addition	13,847.9 (17,384.22)
9.	Empagliflozin addition	31,004 (17,384.2)
10.	Canagliflozin addition	28,301.4 (17,384.22)

The median annual acquisition cost of drug therapy for diabetes per patient was found to be Indian national rupee (INR) 8,964.4 (1 USD = ~INR 75) as per the original prescriptions when calculated using median retail prices calculated for all the formulations of drugs prescribed for diabetes. Upon substituting one of the SGLT-2 inhibitors for the other OADs in the regimen, the cost increased to INR 12,265 (increase by 36.8%) for dapagliflozin, and INR 26,718 and INR 29,419 (increase by ~200%), respectively, for canagliflozin and empagliflozin. When dapagliflozin, canagliflozin, and empagliflozin were added to the original prescriptions, the median annual cost of prescription for diabetes increased slightly to INR 13,848, 28,301, and 31,004, respectively. Upon further substituting sitagliptin/linagliptin as well in addition to the three SGLT-2 inhibitor additions, the annual median cost of prescription came out to be much higher - INR 24,100-41,000 (169-360% increase).

The key patient characteristics, demographics, and results of CVD-REAL 2 (Comparative Effectiveness of Cardiovascular Outcomes in New Users of SGLT-2 Inhibitors), EMPA-REG (Empagliflozin Cardiovascular Outcome Event Trial in Type 2 Diabetes Mellitus Patients-Removing Excess Glucose), DECLARE-TIMI (Dapagliflozin Effect on Cardiovascular Events-Thrombolysis in Myocardial Infarction), and CANVAS (Canagliflozin Cardiovascular Assessment Study) are compared in Table 4. To note, patients in the EMPA-REG trial included only patients with prior ASCVD, while the CANVAS, DECLARE-TIMI, and CVD-REAL 2 studies had also recruited patients without ASCVD - those with risk factors (Table 4).

**Table 4:** Patient characteristics and hazard rates of outcomes in key SGLT-2 inhibitor CV outcomes studies. SGLT-2 = sodium-glucose cotransporter-2; CV = cardiovascular; CVD-REAL 2 = Comparative Effectiveness of Cardiovascular Outcomes in New Users of SGLT-2 Inhibitors; EMPA-REG = Empagliflozin Cardiovascular Outcome Event Trial in Type 2 Diabetes Mellitus Patients-Removing Excess Glucose; DECLARE-TIMI = Dapagliflozin Effect on Cardiovascular Events-Thrombolysis in Myocardial Infarction; CANVAS = Canagliflozin Cardiovascular Assessment Study; ACD = all-cause death; HHF = hospitalization for heart failure; MI = myocardial infarction; 3P-MACE = three-point major adverse cardiac event; 4P-MACE = four-point major adverse cardiac event.

Variable	CVD-REAL 2	EMPA-REG	DECLARE-TIMI	CANVAS
Study population (n)	470,128	7,020	17,160	10,142
SGLT2 inhibitors group (n)	235,064	4,687	8,582	5,795
Non SGLT2 inhibitors/placebo group (n)	235,064	2,333	8,578	4,347
Intervention	Dapagliflozin (74.7%), empagliflozin (9%), other drugs (16.3%)	Empagliflozin	Dapagliflozin	Canagliflozin
Mean/median follow-up duration (in years)	1	3.1	4.2	2.4
Mean age of patients (in years)	56.7	63.1-63.2	63.9-64	63.3
Prior cardiovascular disease	27%	99%	41%	66%
Outcomes with their hazard rates per 1000 patient-years for SGLT2 inhibitors vs non-SGLT2 inhibitors				
ACD	8.0 vs. 13.0	19.4 vs. 28.6	15.1 vs. 16.4	17.3 vs. 19.5
HHF	12.3 vs. 14.8	9.4 vs. 14.5	6.2 vs. 8.5	5.5 vs. 8.7
Composite of ACD or HHF	19.1 vs. 25.1		-	-
MI (Fatal or Nonfatal)	4.5 vs. 5.6	16.8 vs. 19.3	11.7 vs. 13.2	11.2 vs. 12.6
Stroke (fatal or nonfatal)	13 vs. 16.2	12.3 vs. 10.5	6.9 vs. 6.8 (ischemic)	7.9 vs. 9.6
3P-MACE	-	37.4 vs. 43.9	22.6 vs. 24.2	26.9 vs. 31.5
3P-MACE plus hospitalization for unstable angina (4P-MACE)	-	46.4 vs. 52.5	-	-
Composite of cardiovascular death or HHF	-	19.7 vs. 30.1	12.2 vs. 14.7	16.3 vs. 20.8
Renal composite outcome	-	-	3.7 vs. 7	5.5 vs. 9
Progression of albuminuria	-	-	-	89.4 vs. 128.7
Death from cardiovascular causes	-	12.4 vs. 20.2	7 vs. 7.1	11.6 vs. 12.8

Incremental costs with SGLT-2 inhibitors per event prevented cost-effectiveness

Table 5 shows the results of calculating the ICERs with substitution and addition of different SGLT-2 inhibitors (with/without further substitution of DPP-IV inhibitors) to the prescribed regimens. The number of patients needed to be treated for the follow-up duration of each respective study and the total years of treatment needed to prevent one event are also displayed with each hypothetical addition or substitution of the drug (Table 5).

**Table 5:** Incremental costs per event prevented (ICERs) with SGLT-2 inhibitors. n = number of patients needed to be treated (up to the follow-up duration of the respective study); T = number of patient-years of treatment required per event prevention; ICER = incremental cost-effectiveness ratio; SGLT-2 = sodium-glucose cotransporter-2; ACD = all-cause death; HHF = hospitalization for heart failure; MI = myocardial infarction; 3P-MACE = three-point major adverse cardiac event.

Therapy change and name of study	Incremental cost per ACD prevented in INR (with n, T)	Incremental cost per HHF prevented in INR (with n, T)	Incremental cost per 3P-MACE prevented in INR (with n, T)	Incremental cost per MI prevented in INR (with n, T)	Incremental cost per stroke prevented in INR (with n, T)	
						Dapagliflozin addition						
CVD-REAL 2	976,700 (200, 200)	1,953,400 (400, 400)	NA	4,439,101.5 (909, 909)	1,523,652 (312, 312)	
DECLARE-TIMI	37,563,882 (1831, 7692)	2,124,322 (103, 435)	3,052,187.5 (156, 625)	3,257,294.5 (159, 667)	(No benefit)	
Dapagliflozin substitution						
CVD-REAL 2	660,020 (200, 200)	1,320,040 (400, 400)	NA	2,999,790.9 (909, 909)	1,029,631.2 (312, 312)	
DECLARE-TIMI	25,384,369.2 (1831, 7692)	1,435,543 (103, 435)	2,062,562.5 (156, 625)	2,201,166.7 (159, 667)	(No benefit)	
Dapagliflozin and sitagliptin/linagliptin substitution						
CVD-REAL 2	3,027,600 (200, 200)	6,055,200 (400, 400)	NA	13,760,442 (909, 909)	4,723,056 (312, 312)	
DECLARE-TIMI	1,164,41,496 (1831, 7692)	6,585,030 (103, 435)	9,461,250 (156, 625)	10,097,046 (159, 667)	(No benefit)	
Empagliflozin addition						
EMPA-REG	2,395,608.7 (35, 109)	4,321,490.2 (63, 196)	3,390,707.7 (50, 154)	8,815,840 (129, 400)	(No benefit)	
Empagliflozin substitution						
EMPA-REG	2,223,326.09 (35, 109)	4,010,705.9 (63, 196)	3,146,861.5 (50, 154)	8,181,840 (129, 400)	(No benefit)	
Empagliflozin and sitagliptin/linagliptin substitution						
EMPA-REG	3,519,938.1 (35, 109)	6,329,430 (63, 196)	4,973,123.5 (50, 154)	12,917,204 (129, 400)	(No benefit)	
Canagliflozin addition						
CANVAS	8,789,531.8 (126, 454.5)	6,042,803.1 (87, 312.5)	4,203,689.1 (60, 217)	13,812,121.4 (198, 714)	11,374,688.2 (163, 588)	
Canagliflozin substitution						
CANVAS	8,069,818.2 (126, 454.5)	5,548,000 (87, 312.5)	3,859,478.3 (60, 217)	12,681,142.9 (198, 714)	10,443,294.1 (163, 588)	
Canagliflozin and sitagliptin/linagliptin substitution						
CANVAS	13,449,564 (126, 454.5)	9,247,500 (87, 312.5)	6,421,464 (60, 217)	21,128,688 (198, 714)	17,400,096 (163, 588)	

The total incremental costs needed overall to prevent one event of all-cause death (ACD) is INR 660,020, simulating the data from CVD-REAL 2; this would require 200 patients (n) to be treated for one year (T = 200 patient-years of treatment). But a much higher incremental cost of INR 25,384,369.2 is found with the DECLARE-TIMI data with dapagliflozin substitution, requiring 1,831 patients to be treated for 4.2 years or 7,692 patient-years of treatment. These costs become INR 2,223,326.1 with empagliflozin substitution and INR 8,069,818.2 with canagliflozin substitution, requiring 109 and 454.5 patient-years of treatment, respectively.

The incremental cost for prevention of hospitalization for heart failure (HHF) with dapagliflozin substitution is INR 1,320,040-1,435,543; INR 4,010,705.9 with empagliflozin substitution and INR 5,548,000 with canagliflozin substitution to be borne over 196-435 patient-years of treatment as per different study data. To prevent a three-point major adverse cardiac event (3P-MACE) (composite of cardiac death, non-fatal MI, and non-fatal stroke), an additional treatment cost of INR 2,062,562.5 would be needed over 625 patient-years of treatment with dapagliflozin substitution (as per DECLARE-TIMI), and INR 3,146,861.5 and INR 3,859,478.3 with empagliflozin and canagliflozin, respectively, with 154-217 patient-years of treatment. Higher incremental costs are needed to prevent an event of MI, whereas, for stroke, INR 1,029,631.2 over 312 patient-years of treatment was the incremental cost with dapagliflozin (CVD-REAL 2).

Similarly, incremental costs for various outcomes were calculated and found to be relatively higher for the addition of the SGLT-2 inhibitors in the range of INR 2 million to INR 14 million, requiring different patient treatment years, except with dapagliflozin where ICERs were lesser for ACD and stroke prevention as per CVD-REAL 2 data, but higher for ACD if considering the DECLARE-TIMI data. The ICERs were even more significantly higher when SGLT-2 inhibitors were substituted along with further substitution with sitagliptin/linagliptin in the range of INR 3 million to 21 million (with the exception of ACD with dapagliflozin where it increased up to INR 116.4 million).

## Discussion

Upon reviewing all leading diabetes treatment guidelines, it is clear that there are definite recommendations and rationale for using SGLT-2 and DPP-IV inhibitors as the preferred add-on OADs in T2DM patients with established CVD, CKD, HF, or with multiple risk factors. But the cost implications of using these drugs in the Indian population have to be carefully addressed.

Our results show that the annual cost of drug treatment per patient is substantial (approximately INR 9,000) among patients with T2DM having a CV, renal disease, or at high risk. Data from a systematic review has shown that direct and indirect medical costs of diabetes care per patient in India range from INR 8,822-45,792 and INR 3,949-18,707, respectively, in different regions of the country [11]. These combined costs are up to 10-40% of the annual per capita income of Indian citizens. The burden is further worsened by the economic disparity prevalent in the country. Statistics show that there has been a two-fold increase in the number of people living below the poverty line (less than USD 2/INR 145 per day in purchasing power parity) in India due to the coronavirus disease 2019 (COVID-19) pandemic [12]. Indians have to bear most of the financial burden of health care (>50%) out of their own pockets in absence of adequate government support or insurance coverage [13].

At the same time, the burden of cardiovascular and renal complications among Indian diabetics further complicates the picture, placing additional financial burden. Patients with T2DM are at a two-three folds higher risk of developing CVD, contributing to 40% of deaths. They also have a high rate of non-fatal CV events like HHF, which account for up to 33% of such events. Asians with T2DM also have a higher incidence of ischemic stroke and renal disease [10,14-16]. With such a burgeoning burden, it is critical to identify, prevent, and manage CV complications in patients with T2DM [17]. T2DM patients have several CV risk factors, including high blood pressure (BP), dyslipidemia, and albuminuria. Controlling several risk factors has been shown to reduce cardiovascular morbidity and mortality [18]. Looking at data from India, studies like the CUPS (Chennai Urban Population Study) and A1chieve study have shown a high prevalence of CAD (>20%), and risk factors such as hypertension, obesity, and dyslipidemia (23-27%) among patients with T2DM. CV complications are most common after neuropathy in Indian diabetics [19,20]. Several other small-scale epidemiological studies collectively corroborate the significant existing burden of cardiovascular and renal complications in Indian diabetics.

This is where the role of drugs like SGLT-2 inhibitors and GLP-1 RAs comes. Among these, the glucagon-like peptide (GLP) analogs are seriously restricted in their usage due to being injectable, and SGLT-2 inhibitors offer more benefits in HF patients, better tolerability, and once‐daily oral administration [6]. The DPP-IV inhibitors sitagliptin and linagliptin showed non-inferiority in major adverse cardiac event (MACE) risk compared to placebo arms in the CVOTs, but saxagliptin and alogliptin increased the risk of higher HF hospitalizations [21,22]. In contrast, the large CVOTs of SGLT-2 inhibitors (EMPA-REG - empagliflozin, DECLARE-TIMI - dapagliflozin, and CANVAS - canagliflozin) have all demonstrated substantial benefits on various types of CV outcomes, albeit to a variable extent on different parameters. A meta-analysis of the CVOTs of these three SGLT-2 inhibitors indicated a substantial reduction in MACE and hospitalization for HF, as well as a delay in the development of renal complications [23]. Additionally, now there is substantial RWE available from large-scale studies like the CVD-REAL series, EASEL (Evidence for Cardiovascular Outcomes With Sodium-Glucose Cotransporter 2 Inhibitors in the Real World), and OBSERVE-4D, involving close to one million patients, which attests to the benefits observed in the CVOTs. Reasons for such beneficial effects with these drugs have been explored. Their mechanism of action is independent of β‐cell function and insulin resistance. Putative mechanisms for cardio-renal benefits include diuretic and natriuretic effects, positive effects on BP, cardiac load and arterial compliance, reduced body fat, anti-inflammatory effects, reduced sympathetic renin-angiotensin-aldosterone system (RAAS) activity, and increased hematocrit [6,24].

In such a scenario, the implementation of international guidelines like the ADA standards of care supported by Asian consensus recommendations, and even the national guidelines of India, in clinical practice become significant. Oral SGLT-2 inhibitors with CV and renal benefits should ideally be prescribed in the high-risk or at-risk T2DM patients but this is counterbalanced by their high costs, making affordability a key parameter against their prescription. Several studies show low prescribing rates in India for these drugs, and their use as the third- or fourth-line drug [7,8]. Thus, it becomes important to actually try and understand what is the extent of the additional costs with the use of SGLT-2 inhibitors when seen in contexts of the additional benefits, essentially their cost-effectiveness. While a simulation methodology like this is not ideal, the lack of strictly Indian outcome data, combined with expert consensus supporting the applicability of the recommendation regarding early use of SGLT-2 inhibitors in the Asian population for these subsets of patients, the inclusion of Asian patients in CVOTs plus the corroborating results from real-world studies make the extrapolation of these outcomes data to our study population rational and justified. Generating long-term real outcomes data would be ideal, but is not feasible in most situations.

So, looking at the ICERs (essentially the costs per event prevented) generated for the various CV events, it can be deduced that among the three commonly used SGLT-2 inhibitors available in India, dapagliflozin is the most cost-effective for the prevention of 3P-MACE (a composite of CV death, non-fatal stroke, and non-fatal MI), HHF, and MI while benefits for prevention of ACD and stroke are not so clear-cut because of discordant results from the real-world study and the CVOT. This is driven largely by the recent development in India whereby dapagliflozin has become the first SGLT-2 inhibitor to go off-patent, and thus multiple generic versions (branded generics) are now available at significantly lower prices than the other drugs of its class. The average price of a dapagliflozin tablet is around INR 15 in the Indian market compared to ~INR 50 or more for empagliflozin and canagliflozin. At the same time, the number of patient-years of treatment required with dapagliflozin is higher than that with empagliflozin for these outcomes and also more than canagliflozin for most outcomes. In simple terms, it means that while the CV benefits can be obtained at lower costs overall (to the healthcare system as a whole including the individual) with dapagliflozin, it would require longer periods of treatment or a greater number of patients to be treated to realize these benefits in terms of reduction of the various CV events.

Like our simulatory exercise, if not already prescribed, SGLT-2 inhibitors can be substituted for one of the OADs being given in the existing drug regimen, barring metformin (in the order of CV safety - pioglitazone > SU > DPP-IV inhibitors). The strategy of adding an SGLT-2 inhibitor to an existing drug regimen would obviously provide additional glycemic control; it will also increase the additional costs and pill burden. In multidrug regimens, we can also substitute a DPP-IV inhibitor with proven CV safety, namely, sitagliptin or linagliptin instead of other drugs; both these gliptin drugs cost approximately the same in India presently.

Now whether these incremental costs can be considered really "cost-effective" can be a very contentious issue, especially seen in the Indian context. Our analysis indicates that the best ICERs are seen if we consider the outcomes data from the real-world study (CVD-REAL 2) in which approximately 75% of patients received dapagliflozin for one year on an average. The incremental costs with dapagliflozin substitution for preventing one event of ACD, HHF, or stroke ranges from approximately INR 660,000 to 1,320,000 (as per CVD-REAL 2 data), which seems quite reasonable. These are not individual costs of treatment; this is the overall cost that will be incurred by the healthcare system for a group of individuals, whether borne out of pocket by patients, government, or insurance. But we also have to look at the number of people needed to be treated to achieve this, for a period equal to the follow-up duration of the reference study (one year for this study); this number varies from 200 to 400 in this case. However, these incremental costs increased considerably for dapagliflozin for the prevention of MI, and when data from the CVOT (DECLARE-TIMI) are considered especially for ACD. The number of patient-years of treatment required per event reduction also varies considerably and needs to be kept in mind.

Similarly, the costs are incrementally higher for prevention of these events with empagliflozin and canagliflozin, with variable numbers needed to treat (for follow-up durations of 3.1 or 2.4 years, respectively) or a number of patient treatment years required, which are actually favorable for empagliflozin; the results are best for ACD and 3P-MACE (109, 154 years). Understandably, when DPP-IV inhibitors are also substituted in the regimens, where applicable, the incremental cost rises further up to the extent of INR 8-20 million per event prevention for stroke and MI with canagliflozin and empagliflozin.

Now to say whether spending INR one million or 20 million over a period of one to four years of follow-up in 35-200 patients (taking the different study data into consideration) is really worth preventing a major CV event is quite subjective. In crude terms, how much is a middle or old-aged diabetic patient’s life worth? Or what can be considered a reasonable expenditure to reduce the chances of an event like MI, stroke, or HHF from happening in the high-risk group of patients is a very subjective matter, to be seen in many contexts and perspectives.

We also need to look at the costs associated with the management of these adverse CV or renal outcomes in India if they do occur eventually in these high-risk T2DM patients, because this is exactly the expenditure that could be prevented by the use of SGLT-2 inhibitors (with/without DPP-IV inhibitors). In a study assessing the healthcare expenditure of patients with HF in India, it was found that the average total expenditure per patient during a two-year follow-up period was INR 133,663, out of which >60% was out-of-pocket expense [25]. Another study reported that out-of-pocket cost per acute MI was USD 480 (approximately INR 35,000), with the cost being much higher among non-insured patients (58.1%) [26]. Similarly, the mean overall cost (direct and indirect costs) of stroke in a six-month period per patient was found to be INR 80,612 a few years ago [27]. Moreover, the cost of hospitalization for CVDs becomes five times higher in the private setups as compared to the public.

If we consider their therapeutic profile as OADs, the glycemic control offered by SGLT-2 inhibitors as compared to other add-on OADs is quite comparable and even better in most instances, not to mention the additional benefits of weight and blood pressure reduction. The drugs have a very low propensity to cause hypoglycemia but do have some adverse effects, most notably the increased frequency of urination, genital mycotic infection, and risk of urinary tract infections. However, these problems are generally tolerable, preventable, or treatable. The risk of euglycemic diabetic ketoacidosis (DKA) was observed but has a very low incidence. The increased risks of bone fractures and lower extremity amputations with canagliflozin observed in the CANVAS trial have not been corroborated further in large-scale real-world studies [6,28].

In addition to the aforementioned and analyzed CV benefits, SGLT-2 inhibitors also offer renal benefits. In continuation of the benefits already displayed in earlier CVOTs, more recent trials such as CREDENCE (Canagliflozin and Renal Endpoints in Diabetes With Established Nephropathy Clinical Evaluation) have shown significantly reduced risk of disease progression or death from renal or CV disease (by 30%) in diabetic patients with CKD [1,6]. If we focus on dapagliflozin, the cheapest SGLT-2 inhibitor available in India with considerable established CV benefits, the results from the newer trials have been further encouraging even in non-diabetic patients. The DAPA-CKD (Dapagliflozin and Prevention of Adverse Outcomes in Chronic Kidney Disease) trial data show that the drug reduces the risk of decline in estimated glomerular filtration rate (eGFR), end-stage renal disease (ESRD), or death from renal or CV causes (as a composite outcome) in patients with CKD with/without diabetes [29]. Results from the DAPA-HF (Dapagliflozin and Prevention of Adverse Outcomes in Heart Failure) trial show that the drug significantly reduces the risk of worsening of HF or death from CV causes among patients with HF with reduced ejection fraction with/without diabetes [30].

An important aspect of this whole scenario is "patient preferences," which need to be taken into account through the process of shared decision-making. In simple terms, they need to be informed about how drugs like SGLT-2 inhibitors bring down various event risks, what is the extent of this risk reduction, and how does it translate to an individual risk reduction level. Patient choice cards and graphical demonstration of risks of events with or without SGLT-2 inhibitors should be attempted. Eventually, it does come down to the affordability of the patients, making selective SGLT-2 inhibitor prescription perhaps the way to go, both with the drug selection especially with dapagliflozin, and with appropriate patient selection. Dapagliflozin generics can change the OAD prescribing landscape significantly in a country like India, proving to be the relatively cost-effective SGLT-2 inhibitor - the caveat being more patients to be treated for longer durations to see the CV benefits. Another drug remogliflozin has also entered the Indian market, priced quite competitively around the same bracket. However, long-term outcomes data are currently not available for remogliflozin, and the drug requires daily dosing twice. Finally, if the healthcare authorities consider the cost-effectiveness of these drugs worthy enough, their accessibility and affordability can be further improved by bringing them under price control, making them available at Jan Aushadi (generic drugs) stores or at subsidized rates.

Limitations

The study has inherent limitations due to its design as a simulatory and not an actual cost-effectiveness study, using extrapolated outcomes data from previously well-conducted studies instead of originally real-world outcomes data. While there is adequate justification to support the utility of such extrapolated data, it cannot be considered completely applicable in the Indian scenario. India does not have official data on average wholesale prices (AWP) of drugs available in the market like the USA, so we have to rely on obtaining average drug costs by using all the market prices of brands available in the market through various commercial resources. The sample size of prescriptions of high-risk patients used for making hypothetical changes is quite limited, being from a single center. Data from a registry would be ideal to conduct this type of analysis on a large scale, but again we lack such electronic databases in India. Finally, this study is aimed at only providing an estimate about the incremental costs involved and numbers needed to treat patients to prevent CV outcome events in the Indian context, and does not provide conclusive cost-effectiveness data regarding the use of SGLT-2 or DPP-IV inhibitors.

## Conclusions

Looking at the overall evidence available, and the broader or long-term cost-effectiveness of a treatment option like dapagliflozin, it appears that the time has come to implement the recommendations of considering SGLT-2 inhibitors as the early and preferred therapeutic option for the primary prevention of events like heart failure hospitalization and secondary prevention of CVD in patients with T2DM with high-risk factors.
